# Patient-specific 3D-printed HDR surface brachytherapy for basal cell carcinoma in older patients: prospective feasibility and imaging response

**DOI:** 10.1093/oncolo/oyag206

**Published:** 2026-05-26

**Authors:** Piotr Sobolewski, Mateusz Koper, Michal Poltorak, Pawel Banatkiewicz, Malgorzata Kolos, Lukasz Poltorak, Maciej Szwast, Irena Walecka

**Affiliations:** The National Medical Institute of the Ministry of the Interior and Administration, Woloska 137, 02-507 Warsaw, Poland; Chair and Clinic of Dermatology and Pediatric Dermatology, Centre of Postgraduate Medical Education, 02-507Warsaw, Poland; The National Medical Institute of the Ministry of the Interior and Administration, Woloska 137, 02-507 Warsaw, Poland; The National Medical Institute of the Ministry of the Interior and Administration, Woloska 137, 02-507 Warsaw, Poland; The National Medical Institute of the Ministry of the Interior and Administration, Woloska 137, 02-507 Warsaw, Poland; The National Medical Institute of the Ministry of the Interior and Administration, Woloska 137, 02-507 Warsaw, Poland; Electrochemistry at Soft Interfaces Team, Department of Inorganic and Analytical Chemistry, Faculty of Chemistry, University of Lodz, Tamka 12, 91-403 Lodz, Poland; Department of Chemical and Process Engineering, Warsaw University of Technology, Warynskiego 1, 00-645 Warsaw, Poland; The National Medical Institute of the Ministry of the Interior and Administration, Woloska 137, 02-507 Warsaw, Poland; Chair and Clinic of Dermatology and Pediatric Dermatology, Centre of Postgraduate Medical Education, 02-507Warsaw, Poland

**Keywords:** geriatric patients, basal cell carcinoma, high-dose brachytherapy, 3D-printed applicator, dermoscopy

## Abstract

**Background:**

Basal cell carcinoma (BCC) is common in older adults, many of whom are poor surgical candidates, especially for curved and cosmetically sensitive facial sites. High-dose-rate (HDR) surface brachytherapy is effective, but standard flat applicators may fit poorly and increase dose heterogeneity. Patient-specific 3D-printed applicators could improve conformity, yet geriatric BCC data are limited.

**Objective:**

To assess the feasibility and 12-month outcomes of personalized 3D-printed HDR surface brachytherapy in geriatric patients with BCC.

**Methods:**

In this prospective single-arm study, 15 patients aged ≥65 years with histologically confirmed BCC (predominantly facial) who were unsuitable for surgery received HDR surface brachytherapy using custom 3D-printed applicators. Treatment delivered 51 Gy in 17 fractions with CT-based planning. Repeat CT imaging evaluated applicator fit/reproducibility and target coverage. Acute skin toxicity was graded by RTOG. At 12 months, response was assessed clinically and dermoscopically; cosmesis and quality of life (Dermatology Life Quality Index [DLQI]) were recorded.

**Results:**

All patients completed treatment. Positioning was highly reproducible (mean setup error <1 mm) with >90% target dose coverage. At 12 months, all lesions achieved complete remission, with no local recurrence on dermoscopy. Acute reactions were mostly mild (73% grade 1), with 13% grade 2 and 13% grade 3; no grade 4 toxicity or serious adverse events occurred. Cosmesis was favorable, and DLQI improved (mean, 10.5 pre-treatment vs 2.9 at 1 year; *P* < .01).

**Conclusions:**

Personalized 3D-printed HDR surface brachytherapy appears feasible and well tolerated in this small, single-center cohort of older patients with BCC, with favorable 1-year local control and quality-of-life improvement when surgery is contraindicated. However, the limited sample size and single-center design represent limitation, and the observed benefit should be regarded as suggestive rather than definitive.

Implications for Practice Personalized 3D-printed HDR surface brachytherapy may offer a feasible, noninvasive option for older patients with basal cell carcinoma who are poor surgical candidates, particularly those with lesions on complex facial anatomy. The technique enables precise, reproducible dose delivery and favorable early local control and cosmetic outcomes, while dermoscopic monitoring can support noninvasive follow-up. However, these findings are derived from a small, single-center cohort and should be viewed as preliminary, underscoring the need for larger studies before broad implementation in routine geriatric skin cancer care.

## Introduction

Non-melanoma skin cancers (NMSCs), comprising primarily basal cell carcinoma (BCC), represent the most prevalent malignancies worldwide, with a particularly high burden among geriatric patient. The incidence of BCC rises steeply with age due to cumulative ultraviolet (UV) exposure, immunosenescence, and comorbidities that may impair effective skin repair.^1^ Although these tumors are typically less aggressive than melanoma, untreated or recurrent lesions can lead to pain and cosmetic defects, especially when located in areas such as the face, ears, and periorbital region.^2^

Surgical excision remains the standard of care for most NMSC cases; however, this approach is not universally applicable.^3^ Moreover, its applicability is often limited in geriatric patients, who may present with multimorbidity, frailty, polypharmacy, anticoagulant use, or reduced wound-healing capacity. These factors increase perioperative risk and make surgery less suitable for older adults, particularly when the expected cosmetic or functional outcome is poor. In these scenarios, radiotherapy (RT) offers a noninvasive, organ- and tissue-sparing alternative that has demonstrated high rates of local control.^4^

Hypofractionated RT has emerged as an effective, organ-preserving treatment option for NMSC, particularly for patients who are not ideal surgical candidates.^5^^,^^6^ By delivering higher doses per fraction over a limited number of sessions, hypofractionation offers significant advantages in terms of patient convenience, treatment efficiency, and reduced burden on healthcare systems—factors especially relevant in elderly or comorbid patients.^7^

Among available modalities, high-dose-rate (HDR) brachytherapy is frequently used due to its superficial penetration, which is well-suited for cutaneous tumors. However, its efficacy relies heavily on the precise delivery of dose to the tumor-bearing surface.^8^ Achieving uniform dose distribution across irregular or anatomically complex areas—such as the nose, ear, or scalp—remains a significant technical challenge. Traditional flat applicators are often ill-fitting on curved or mobile skin, leading to air gaps, poor conformity, and dose heterogeneity. These factors can compromise tumor coverage, resulting in areas of underdosage at tumor margins and increased risk of local recurrence–issues particularly important in older patients whose skin at baseline may be thinner, more elastic, and more prone to treatment-related toxicity.

Brachytherapy, particularly surface techniques using HDR sources, has also shown excellent outcomes in the treatment of superficial skin cancers.^9^ This modality involves the placement of radioactive sources close to or in direct contact with the lesion, allowing for steep dose gradients and highly localized treatment.^10^ Nevertheless, brachytherapy typically requires specialized applicators or molds tailored to individual anatomy.^11^ Conventional fabrication methods for such devices are labor-intensive, time-consuming, and often lack reproducibility—barriers that have limited widespread adoption of brachytherapy in routine dermatologic oncology practice, especially in settings caring for large numbers of geriatric patients.

In brachytherapy approaches, ensuring accurate, reproducible, and anatomically conformal dose delivery is essential for treatment success. Recent advances in three-dimensional (3D) printing have opened new possibilities for overcoming these limitations through on-demand creation of patient-specific applicators. By enabling the rapid, low-cost fabrication of individualized bolus and applicator devices that conform precisely to patient anatomy, 3D printing enhances dosimetric accuracy and reproducibility.^12^ This patient-specific approach is particularly valuable in treating lesions located on complex anatomical contours, where standard bolus methods may fall short.

While the technological feasibility of 3D-printed applicators has been established in phantom and dosimetric studies,^13^ clinical data, particularly those evaluating integrated imaging endpoints, remain limited. Concurrently, there is a growing need for objective, noninvasive tools to assess tumor response and local control following RT. Traditional clinical inspection may be insufficient to detect subtle residual disease or early recurrence. Advanced skin imaging modalities, such as dermoscopy, videodermoscopy, reflectance confocal microscopy (RCM) and line-field confocal optical coherence tomography (LC-OCT) provide complementary layers of diagnostic information, offering real-time and noninvasive visualization of vascular and cellular changes associated with treatment response.^14^^,^^15^

In particular, RCM allows for near-histologic resolution imaging of the epidermis and superficial dermis, enabling the detection of residual tumor architecture or vascular features indicative of active disease.^16^ When used alongside videodermoscopy, which captures changes in vascular morphology and pigmentation patterns, these modalities offer a robust, multimodal approach to post-RT surveillance.

In this context, we present results of the prospective clinical study combining high-dose, hypofractionated HDR brachytherapy with 3D-printed, patient-specific surface applicators and videodermoscopy for treatment monitoring. In a cohort of 15 patients with histologically confirmed BCC, we evaluate the feasibility of this approach, its dosimetric accuracy, acute toxicity profile, and early treatment response based on both clinical examination and noninvasive skin imaging, as well as patient-reported quality of life assessed using the Dermatology Life Quality Index (DLQI). To the best of our knowledge, this is the first prospective study to assess dermoscopic response patterns after RT delivered with patient-specific 3D-printed applicators, specifically in a geriatric population. This study aims to provide novel clinical evidence supporting the integration of 3D printing and advanced imaging in the personalized management of skin cancer.

## Materials and methods

The objective of this study was to evaluate the dermoscopic characteristics of BCCs in geriatric patients (defined as individuals aged 65 years and older) eligible for brachytherapy, followed by an analysis of the clinical and dermoscopic patterns within the treated area. Clinical assessments were conducted, and macroscopic as well as dermoscopic images of the tumors were obtained for subsequent comparative analysis. Dermoscopic features emerging, regressing, or undergoing modification after exposure to ionizing radiation were examined. In addition, patients’ quality of life before and after RT was assessed using the DLQI questionnaire. All lesions before brachytherapy were confirmed in histopathological examination by the punch biopsy. Standard stains with hematoxylin and eosin were used in all cases, and the specimens were assessed by a qualified and experienced pathologist according to WHO guidelines edition 2023.

Fifteen geriatric patients with a median age of 76 years (SD, 10.2 years) who received HDR brachytherapy between February 2023 and September 2024 were included in the study, according to proper IRB approval. Patients were disqualified from surgery because of advanced age, the poor expected cosmetic or functional outcome of surgery—tumor localization in the central face region, or due to patient’s choice. Tumors were localized in the central facial region in 12 cases, lateral face in 2 cases and back in 1 case.

**Figure 1.**
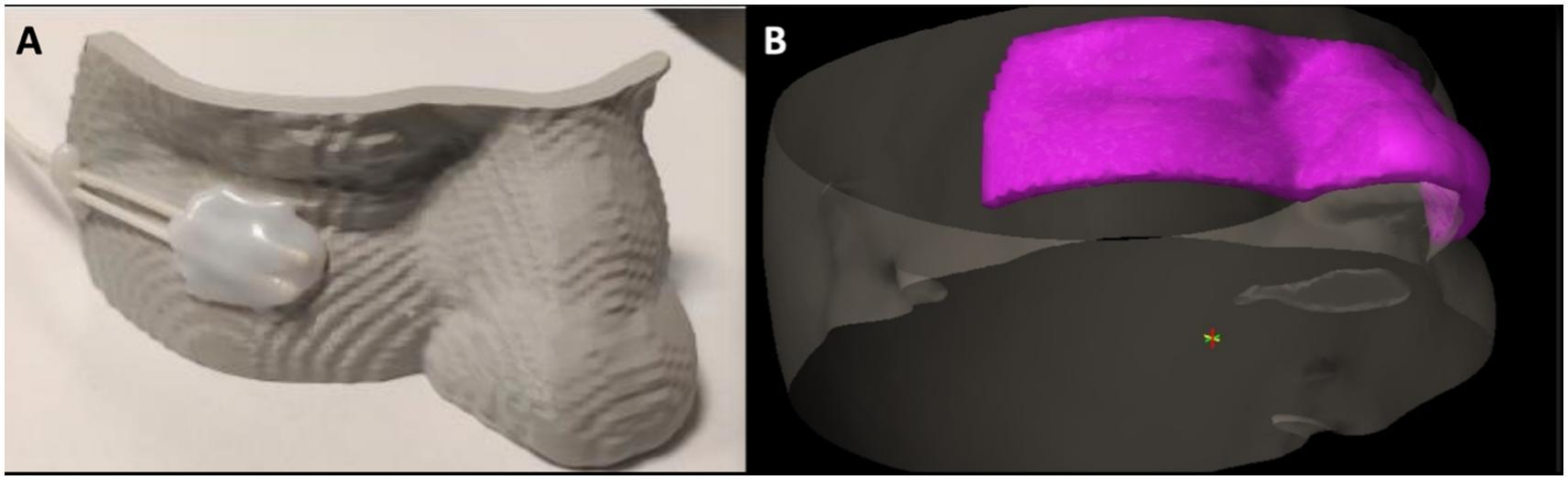
3D-printed applicator (A) and 3D visualization (B).

Patient-tailored applicators were produced using 3D-printing technology with polylactic acid (PLA) filament from Fiberology. The fabrication process was carried out on FDM machines, specifically the Prusa Mini and Prusa iMk3+ models. Individual designs in .stl format were obtained by transforming computed tomography (CT) scans of the skin cancer–affected anatomical site into printable files. These were processed in Prusa Slicer to generate the required g-code. Printing parameters typically included a layer thickness of 0.15 mm, complete (100%) infill, and variable print speeds ranging from 25 to 80 mm/s depending on nozzle trajectory. To reduce the likelihood of surface defects while maintaining print reliability, support structures were defined manually for each device. To investigate the stability of applicator placement throughout treatment, a series of CT studies was performed. The initial scan from the first treatment session was used as the reference dataset. Additional scans were acquired every third brachytherapy fraction with the applicator positioned on the patient–particularly important in elderly patients whose skin is more prone to displacement or wrinkling during treatment. Image registration methods, guided primarily by bony landmarks, were applied to align the scans for comparison. Positional shifts were then determined at several points along the applicator, including the channel endpoints where the radiation source stopped. The thickness of the air gap between the applicator surface and the patient’s skin was also measured at three locations within the treatment region. These evaluations allowed for a detailed characterization of potential deviations in applicator fit and positioning relative to patient anatomy.^17^ An example of a 3D-printed applicator and its corresponding 3D visualization are presented in Figure 1.

HDR brachytherapy was delivered with a total prescribed dose of 51 Gy in 17 fractions. The fractionation scheme was chosen in accordance with established clinical practice for superficial skin lesions, balancing treatment efficacy with the minimization of late radiation toxicity.

Treatment planning was based on CT imaging, which was performed with the patient immobilized and the 3D-printed applicator in place. The CT scans served as the foundation for contouring both the target volume and adjacent organs at risk. On serial CT checks, applicator placement was reproducible with minimal shifts (mean, <1.0 mm, SD, 0.9 mm) and negligible air gaps. The clinical target volume (CTV) was defined as the visible tumor area with an appropriate margin, while the planning target volume (PTV) accounted for possible uncertainties in positioning and applicator placement.^17^

Subsequently, the treatment plan was generated using a dedicated brachytherapy planning system. Dwell positions and times of the ^192Ir source were optimized to achieve conformal dose coverage of the PTV while respecting dose constraints for surrounding healthy tissues. Dose distribution was evaluated through dose–volume histograms (DVHs), ensuring that at least 90% of the prescribed dose encompassed the PTV (D90 ≥ 100%), with hotspot and skin surface doses carefully assessed.

The prescribed regimen of 51 Gy in 17 fractions reflects a balance between adequate tumor control probability and acceptable normal tissue complication probability, and it is consistent with published HDR brachytherapy protocols for non-melanoma skin cancers.

In our study, we also evaluated acute radiation-induced skin reactions using the Radiation Therapy Oncology Group (RTOG) scoring system. The RTOG scale is commonly used to classify the intensity of adverse effects experienced during RT. It establishes uniform guidelines for describing radiation-induced skin toxicity, enabling accurate reporting in clinical practice and facilitating comparisons between different studies or treatment protocols. Within this framework, grade 0 corresponds to the absence of changes relative to baseline. Grade 1 is characterized by mild symptoms such as faint or dull erythema, follicular reactions, partial hair loss, dry desquamation, or reduced sweating. Grade 2 involves more noticeable skin responses, including bright or tender erythema, localized moist desquamation, and moderate edema. Grade 3 reflects severe conditions such as confluent moist desquamation outside skin folds or the presence of pitting edema. The most serious category, grade 4, is defined by the development of ulceration, bleeding, or necrosis.^18^

Clinical evaluation of BCCs was performed according to 16 dermoscopic features at the beginning of the treatment (t1) and one year after the end of the treatment (t2). Digital photographic images were obtained using Medicam 1000 digital dermoscopy camera (MC1000-3-2517, FotoFinder Systems GmbH, Germany, 2022). Presence (1) or absence (0) of the BCC dermoscopic features were described by two different dermoscopists.^19^^,^^20^

Before statistical analysis, we examined the inter-rater reliability (IRR) between two physicians (Observer 1 and Observer 2). All dermoscopic images were evaluated independently and in a blinded manner, with the image order randomized prior to assessment to minimize subjectivity in evaluation. For this purpose, we used Cohen’s kappa coefficient. Subsequently, McNemar’s test was used for between-group differences in dermoscopic features. A value of two-sided *P* < .05 was considered significant. All analyses were performed manually in Excel using standard formulas.

## Results

No deaths occurred during the study. The mean DLQI score decreased significantly from 10.47 ± 2.29 before RT to 2.87 ± 1.30 at 1 year after treatment, reflecting meaningful enhancement of daily functioning and well-being in this older cohort. The difference between pre- and post-treatment scores was statistically significant (*P* < .01). Details are listed in Table 1.

**Table 1.** Survival and patients’ quality of life parameters.

Parameter	T1	T2	*P* value
**Survival**	15	15	1.0
**DLQI**	10.47 ± 2.29	2.87 ± 1.30	**<0.01**

Values are means, ± SD. *P* value by T-test. T1—the start of the experiment (before brachytherapy). T2—the end of the experiment (12 months after brachytherapy). *P* < 0.05 considered significant.

In our cohort, acute radiation-induced skin reactions were evaluated using the RTOG grading system. Most patients (11/15, 73.3%) exhibited grade 1 toxicity, typically characterized by faint erythema or dry desquamation. Two patients (13.3%) presented with grade 2 reactions, while another two (13.3%) developed grade 3 skin toxicity, showing more intense erythema and areas of moist desquamation. In three cases both acute and chronic radiation reactions were observed. No grade 4 toxicity was reported. Overall, the majority of patients experienced only mild radiation-induced skin effects, with severe reactions being uncommon. These findings indicate that HDR brachytherapy with individualized applicators is generally well tolerated in older adults, despite their increased susceptibility to skin fragility and slower healing.

All 15 lesions were observed two times–at the beginning of the treatment (t1) and 1 year after the end of the treatment (t2). The list of 16 dermoscopic features of BCC and the *P* value is reported in Table 2.

**Table 2.** Dermoscopic features of BCCs changes with corresponding *P* value.

Dermoscopic feature	*P* value
**Telangiectasia**	0.250
**Short vessels**	1.000
**Crust/scale**	**0.008**
**Clods (blue, large, clustered)**	0.063
**Clods (white, shiny)**	**0.031**
**Gray dots**	1.000
**Lines (white, perpendicular)**	1.000
**Branched vessels**	0.063
**Serpentine vessels**	**0.016**
**Curved vessels**	0.500
**Polymorphous vessels**	**0.016**
**Blue structureless zone**	1.000
**Pink structureless zone**	0.070
**Erosion**	**<0.001**
**Ulceration**	**<0.001**
2MAY globules*	0.063

* MAY globules = multiple aggregated yellowish globules. *P* < 0.05 considered significant.

At the 1-year follow-up, all treated lesions showed complete clinical response, with no evidence of persistent or recurrent tumor on clinical and dermoscopic examination.

The IRR between Observer 1 and Observer 2, assessed using Cohen’s kappa, was κ = 0.879, indicating an almost perfect level of concordance.^21^

Following brachytherapy, several dermoscopic characteristics of BCC underwent notable modifications, reflecting both tumor regression and therapy-induced alterations in the skin. Statistical analysis revealed that some BCC features significantly decreased in frequency, while others remained relatively unchanged.

Among vascular structures, telangiectasia (*P* = .250), short vessels (*P* = 1.000), curved vessels (*P* = .500), branched vessels (*P* = .063), and polymorphous vessels (*P* = .016) showed variable responses to treatment. Although telangiectatic and short vessels did not change significantly, the presence of serpentine vessels (*P* = .016) and polymorphous vessels (*P* = .016) decreased significantly after therapy, suggesting a reduction in tumor-associated angiogenesis and vascular heterogeneity. The slight decrease in branched vessels approached significance (*P* = .063), which may indicate partial vessel regression secondary to radiation effects.

Regarding structureless zones, blue structureless areas (*P* = 1.000) remained stable, consistent with their possible association with residual pigmentation or fibrosis rather than viable tumor tissue. In contrast, pink structureless zones showed a borderline reduction (*P* = .070), potentially reflecting decreased vascularization and inflammation in regressed lesions.

Among nonvascular features, significant reductions were observed in crusts or scales (*P* = .008), white shiny clods (*P* = .031), and particularly in erosions (*P* < .001), and ulcerations (*P* < .001). These findings suggest progressive re-epithelialization and lesion healing following RT. The decrease in white shiny clods may correspond to reduced stromal reaction or fibrosis, while the disappearance of erosions and ulcerations is consistent with effective tumor resolution and post-treatment regeneration of the epidermis.

Other features such as blue clods (*P* = .063), gray dots (*P* = 1.000), white perpendicular lines (*P* = 1.000), and MAY globules (*P* = .063) did not show statistically significant differences, indicating either post-radiation fibrosis or a scar tissue process.

**Figure 2.**
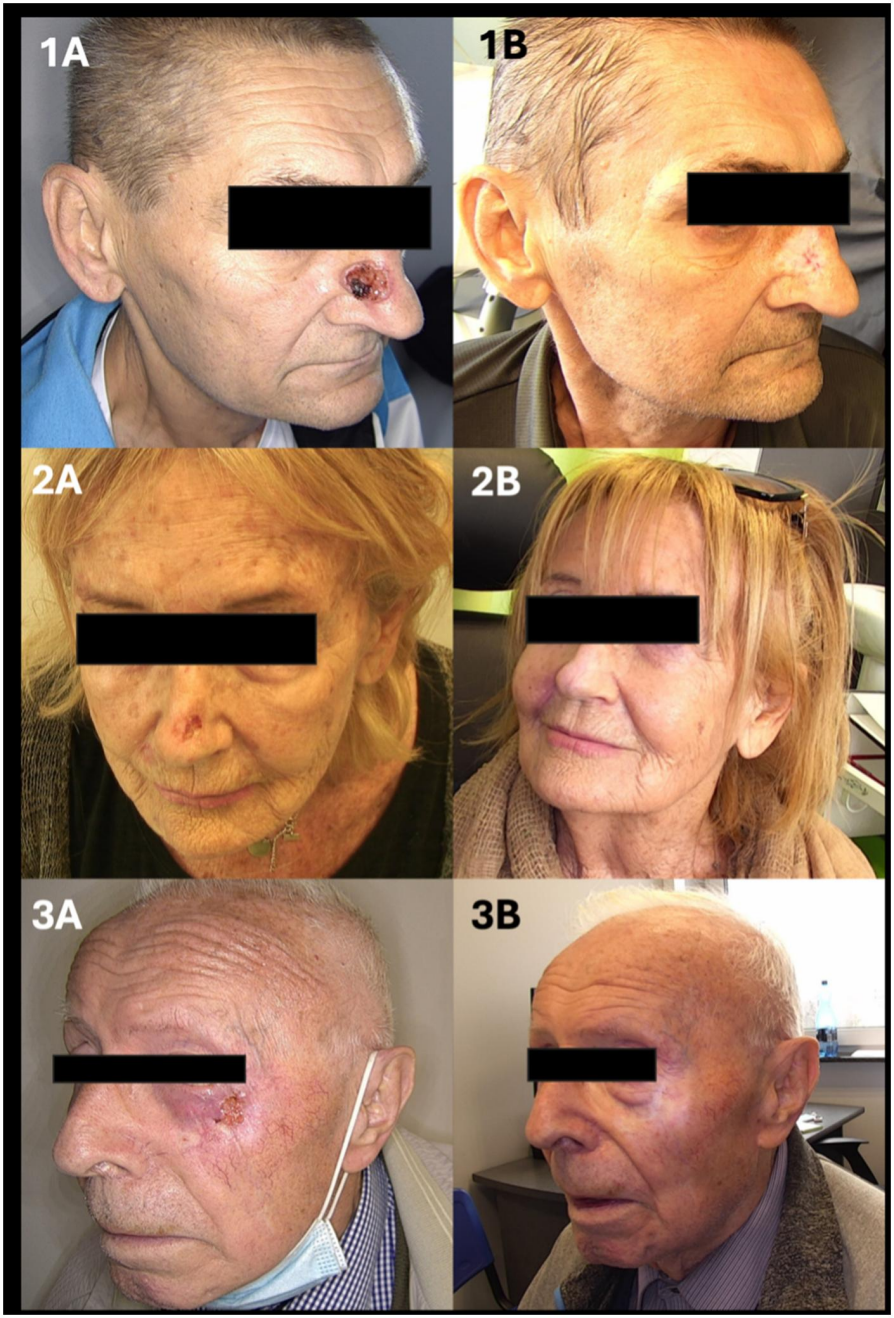
Macroscopic images before brachytherapy (1A, 2A, 3A) and 12 months after brachytherapy (1B, 2B, 3B).

**Figure 3.**
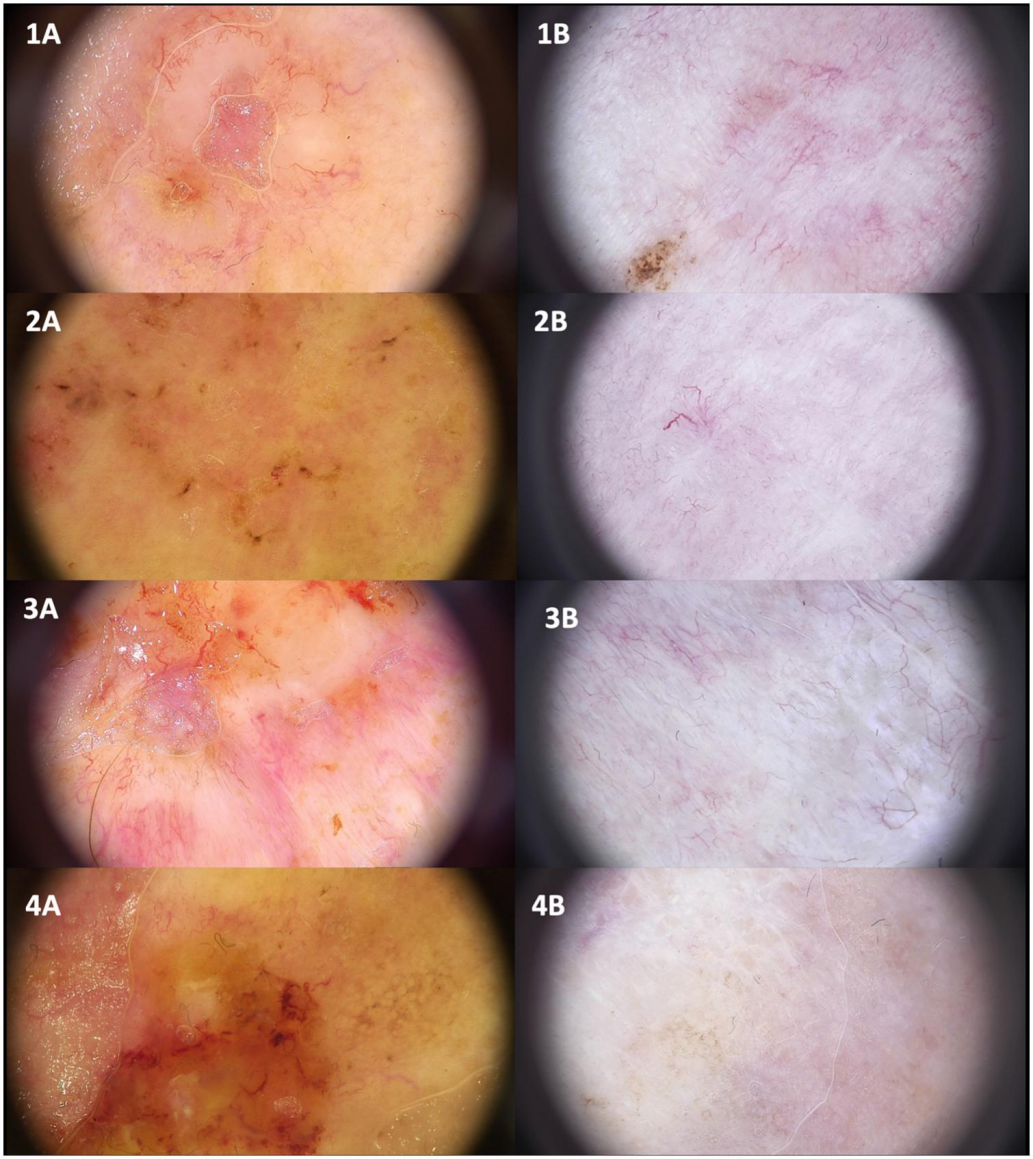
Dermoscopic images before brachytherapy (1A, 2A, 3A) and 12 months after brachytherapy (1B, 2B, 3B).

Overall, the dermoscopic pattern after brachytherapy is characterized by the disappearance of ulcerative and vascular polymorphism features, together with a reduction of scaling and shiny white structures, suggesting successful tumor control and post-radiation reparative processes in the skin. Representative macroscopic (Figure 2) and dermoscopic (Figure 3) images are provided below.

## Discussion

This prospective study demonstrates that HDR delivered with 3D-printed, patient-specific applicators achieves excellent local control of BCC, with minimal toxicity and favorable cosmetic outcomes in geriatric patients. Our findings are consistent with previous reports highlighting the feasibility and accuracy of 3D-printed devices for surface RT,^12^^,^^17^^,^^22^ but extend current knowledge by integrating dermoscopic imaging for objective post-treatment evaluation.

In our cohort, several dermoscopic features commonly associated with BCC showed significant regression after treatment, particularly serpentine and polymorphous vessels. Importantly, erosions and ulcerations also showed significant resolution following treatment. These results align with prior observations that RT induces characteristic regression patterns in BCCs, reflecting effective tumor eradication and re-epithelialization.^23^ Features such as telangiectasia, white, shiny clods, and globules persisted in some cases, suggesting that they may represent post-treatment inflammatory or reparative changes rather than active disease.^24^ In addition, patients’ quality of life, assessed using the DLQI questionnaire, improved significantly after RT.

Our observations align with previous reports describing dermoscopic regression patterns after different therapeutic modalities, including topical imiquimod,^25–27^ photodynamic therapy,^26^ and surgery. However, only a limited number of studies have specifically evaluated dermoscopic changes after RT.^28^ The present study adds to the growing body of evidence supporting dermoscopy as an effective noninvasive monitoring method in oncologic dermatology. Importantly, dermoscopy can reveal subtle vascular or structural changes that may not be clinically apparent, thereby increasing diagnostic confidence during follow-up.

A major advantage of dermoscopy is its accessibility, low cost, and widespread availability compared to more advanced imaging modalities. Nevertheless, it is important to recognize its limitations. While dermoscopy provides valuable surface-level information, it does not allow visualization of deeper tissue layers or cellular-level changes. For this reason, some findings, such as residual pigmentation or vascular alterations, may be challenging to interpret unequivocally. In such cases, more advanced imaging modalities could provide complementary information.

RCM, for example, offers near-histological resolution and has been shown to improve the detection of residual or recurrent tumor following non-surgical treatments for BCC. Although RCM was not applied in the present study, future investigations should incorporate this modality alongside dermoscopy. Such a multimodal approach would allow the correlation of superficial vascular and pigmentary patterns with deeper microarchitectural changes, potentially improving the accuracy of post-radiotherapy monitoring. Combining these tools could not only strengthen diagnostic certainty but also reduce the need for invasive biopsies in ambiguous cases.

The strengths of our study include its prospective design, exclusive focus on an older population, systematic evaluation of a broad range of dermoscopic features, and the innovative application of patient-specific 3D-printed applicators, which ensured accurate and reproducible RT delivery. Nevertheless, certain limitations must be acknowledged. The study cohort was relatively small and derived from a single center, which may restrict generalizability. The follow-up period was limited to 1 year, precluding conclusions regarding long-term recurrence rates. Finally, while dermoscopy allowed us to capture significant treatment-induced changes, the absence of histopathological or RCM correlation at follow-up limited our ability to confirm the absence of microscopic residual disease. An additional consideration is the inherently interdisciplinary nature of this therapeutic pathway. The diagnosis and initial qualification for treatment are typically performed by dermatologists, who play a central role in identifying suitable candidates for RT. Treatment delivery itself requires close collaboration between radiation oncologists and medical physicists, whose expertise ensures precise dosimetry and safe, reproducible use of patient-specific 3D-printed applicators. Following completion of therapy, dermatologists remain essential for long-term follow-up, using dermoscopy to monitor for potential recurrence as well as to screen for new primary BCCs. Such coordinated, multidisciplinary care is crucial to optimize treatment outcomes and ensure comprehensive patient management.

In summary, our findings support dermoscopy as a practical, noninvasive tool for assessing treatment response after RT for BCC in geriatric population. The observed regression of vascular and structural tumor-associated features confirms the clinical efficacy of RT delivered with 3D-printed applicators and highlights the value of dermoscopy in documenting these changes. Future studies should validate these findings in larger, multicenter cohorts with extended follow-up and integrate additional imaging modalities such as RCM to provide a more comprehensive understanding of tumor clearance and tissue remodeling after RT. Such an approach could refine post-treatment surveillance strategies and further personalize the management of non-melanoma skin cancers.

## Conclusions

This prospective, single-center study of 15 older patients with basal cell carcinoma suggests that patient-specific 3D-printed HDR surface brachytherapy is feasible, dosimetrically accurate, and well tolerated, with excellent 12-month local control and favorable cosmetic and quality-of-life outcomes in a geriatric cohort unfit for surgery. The individualized applicators enabled highly reproducible positioning and consistent target coverage, while dermoscopic follow-up revealed marked regression of hallmark BCC features such as serpentine and polymorphous vessels, erosions, and ulcerations, alongside a significant improvement in DLQI scores. These findings support the concept that 3D-printed HDR surface applicators can enhance dose conformity and reduce acute skin toxicity on anatomically complex, cosmetically sensitive facial sites in older adults. Nevertheless, the data must be interpreted with caution due to the small sample size, monocentric design, and limited follow-up of 1 year, which restrict generalizability and preclude conclusions about long-term recurrence rates. Thus, the observed benefit is suggestive rather than definitive, and further prospective, multicenter studies with larger cohorts and extended follow-up are warranted to confirm the efficacy and safety of this approach, as well as to explore the integration of advanced imaging modalities such as RCM or LC-OCT for more precise post-treatment monitoring of BCC regression and tissue remodeling.

## Data Availability

The data that support the findings of this study are available from the corresponding author upon reasonable request.
